# Elucidation of Strain-Dependent, Zinc Oxide Nanorod Response for Nanorod-Guided Fluorescence Intensity

**DOI:** 10.3390/nano12203558

**Published:** 2022-10-11

**Authors:** Johnson Truong, Andrew Stoner, Marion Ryan C. Sytu, T Rizana Tatlock, David H. Cho, Jong-in Hahm

**Affiliations:** Department of Chemistry, Georgetown University, 37th & O Sts. NW., Washington, DC 20057, USA

**Keywords:** zinc oxide nanorod, stress, strain, guided fluorescence, fluorescence detection, optical signal enhancement

## Abstract

In this work, we examine how strain exerted on individual ZnO nanorods (NRs) can influence the fluorescence signals that are emitted from fluorophore molecules and subsequently coupled into and guided along the NR. We elucidate the relationships between the incremental levels of compressive and tensile strain on the NRs and measured fluorescence intensity of a model fluorophore, rhodamine 6G (R6G), as a function of the position on the NRs. We reveal that compressive strain on the NRs leads to a decrease in the guided fluorescence signal, while tensile strain leads to an increase in the fluorescence intensity. Compared to an unstrained state, approximately 35% decrease (increase) in R6G fluorescence intensity was observed from ZnO NRs when they were under compressive strain of −14% (tensile strain of +10%). Further, our systematic acquisition of the incremental addition of uniaxial strain result in a linear relationship of the coupled fluorescence signal and the amount of applied strain. The degree of fluorescence intensification on nanorod ends (DoF), which is a quantitative indicator for the amount of R6G signals coupled into and waveguided to the NR ends compared to those on the main body, also exhibits a linear relationship with strain. These outcomes, in turn, demonstrate that strain alters the waveguiding capabilities of ZnO NRs in a predictable manner, which can be exploited to modulate and optimize fluorescence and other light signals emitted by a nearby source. Considering the wide utility of ZnO NRs in photonics, optoelectronics, and sensors, insights from our study may be highly valuable to effectively controlling and enhancing optical signals from chemical and biological analytes through strain.

## 1. Introduction

Zinc oxide (ZnO) nanomaterials have been extensively utilized as key components in important technological devices such as optoelectronic modules [1,2,3], nanolasers [4,5,6], strain sensors [7,8], biosensors [9,10,11,12,13], and multi-emission waveguides [14]. In particular, ZnO nanorods (NRs) have drawn considerable attention in the broad fields of photonics, biosensing, and light detection due to their exceptional optical properties that stem from the NRs’ reduced dimensionality and high shape anisotropy [4,7,9,14,15]. As it is critical to tune the NRs’ properties in such applications, various approaches have been employed to control the nanomaterials’ optical, electrical, and piezotronic behaviors. They include changing chemical compositions through doping [16,17,18], altering crystalline structures [19,20,21,22], and applying a magnetic field [23]. More recently, mechanical strain has also been used to modulate ZnO properties [24,25,26,27,28,29]. For example, strain-altered ZnO NRs have been shown to alter their Schottky barrier height [25,26] and electrical conductance [27,28,29]. Strain was also used to change the near band-edge emission of ZnO nanowires [30,31,32,33], to shift the lasing modes of ZnO microwires [24], and to alter the phonon frequencies of ZnO NR Raman modes [34]. These past studies demonstrated how optical signals emitted from the ZnO nanomaterial itself such as the band-edge, photoluminescence, and lasing emissions can be modulated by applying stress. Still, ZnO NRs’ applications reach beyond the manipulation of their intrinsic signals and can involve light signals emitted by other sources located in the vicinity of the NRs. However, it is not yet known how individual ZnO NRs under mechanical stress will change their ability to couple and guide light signals emitted from, for instance, fluorophore-conjugated protein molecules bound on the surface of ZnO NRs in a biosensor. 

We have previously reported on the enhanced fluorescence detection capability as well as on the effect of fluorescence intensification on nanorod ends (FINE) that was revealed from individual ZnO NRs coupled with fluorophore-labeled biomolecules [10,11,12,15,35]. The FINE phenomenon refers to highly intensified fluorescence signals observed at the two NR ends relative to those on the NR main body. Its degree of signal enhancements at the ends versus main body can be quantitatively expressed by degree of FINE (DoF). DoF is the ratio of the average intensities measured at the NR ends versus main body positions. We have shown that these signal enhancing capabilities of ZnO NRs originate from the fact that, as a high-quality optical cavity, individual ZnO NRs can efficiently couple biomolecular fluorescence emission into the NR and subsequently waveguide the light to the two ends of the NR [10,11,12,35]. We have since determined key experimental parameters affecting FINE and DoF [10,35]. In these studies, we examined a variety of factors related to the nanomaterial itself as well as those associated with the fluorophores and biomolecules [10,11,12,15,35]. However, potential effects of mechanical strain applied to ZnO NRs on the intriguing effects of FINE and DoF are still not known. Systematic investigations to understand how modulating strain can influence the optical signal guiding effects of individual ZnO NRs will be critical to their applications as biosensors, waveguides, and strain gauges. Hence, such research efforts are highly warranted.

In this work, we elucidate the effects of mechanical strain on the fluorescence signals emitted by a nearby fluorophore source and carried by individual ZnO NRs. We examine single ZnO NRs for the NR-guided fluorescence signals from a model fluorophore of rhodamine 6G (R6G) while the fluorophore-NR assembly, embedded in a polydimethylsiloxane (PDMS) matrix, undergoes varying levels of uniaxial compressive or tensile strain. We successfully ascertain the effect of strain on the fluorescence signals and quantitatively show the relationships between the different types and levels of strain on the NR and the guided R6G fluorescence intensity in terms of DoF. Our study signifies an important new path for effectively modulating optical signals from an extrinsic source through controlling strain on ZnO NRs, which can be particularly beneficial to the applications of ZnO NRs in strain-sensitive fluorescence biosensors and in vivo nanoprobes for highly localized delivery of light signal. ZnO NRs has remarkable optical capabilities previously demonstrated for their background-free and highly sensitive analyte detection through the NR-enabled, optical signal enhancement and spatial localization [10,11,12,15,35]. In this regard, our research efforts may also facilitate future development of entirely new detection platforms that capitalize on combining the newfound capacity of strain-controlled signal enhancement with those NR capabilities already proven.

## 2. Materials and Methods

ZnO NRs were synthesized in a horizontal tube furnace using a chemical vapor deposition method. In brief, 20 nm Au colloids (Ted Pella, Inc., Redding, CA, USA) serving as a growth catalyst were deposited on a Si plate (Silicon Quest International Inc., San Jose, CA, USA). The source material used for the NR synthesis consisted of a 1:2 mixture of 99.999% ZnO and 99% graphite powders (Alfa Aesar Inc., Tewksbury, MA, USA). A quartz boat housing the source material was placed 15 cm upstream from the Si wafer containing the Au catalysts. The home-built horizontal tube furnace was heated to 950 °C for 15 min to 1 h at a rate of 15 °C min^−1^ under a constant Ar flow at a rate of 100 standard cubic centimeters per minute. The as-grown ZnO NRs were sonicated in ethanol and deposited on a clean Si plate. Subsequently, 40 µL of 250 µg mL^−1^ rhodamine 6G (R6G) in ethanol was added to the ZnO NRs/Si plate and incubated for 10 min in a humidity-controlled chamber. The sample was then rinsed with DI water and dried using a stream of nitrogen. Next, the R6G/ZnO NRs/Si plate was placed face up in a petri dish. A PDMS elastomer mixture (Dow Corning; Midland, MI, USA) was prepared, poured into the petri dish, and cured at 80 °C for 24 h. After the curing process was completed, the entire PDMS construct was removed from the petri dish, and the Si plate was carefully peeled off. A top elastomer layer was then fabricated by pouring and curing an additional layer of PDMS solution on top of the R6G/ZnO NRs/PDMS assembly from the previous steps. The resulting construct of R6G/ZnO NRs embedded in a PDMS matrix was subsequently mounted on to a microvice (S.T. Japan USA LLC, Ft. Myers, FL, USA) for the application of compressive and tensile stress. All measurements were conducted under ambient conditions. Our strategy to enclose NRs fully inside a PDMS elastomer can ensure prolonged NR stability by protecting the nanomaterials from any potential environmental and ageing effects. 

Fluorescence measurements were sequentially performed while systematically changing the magnitude and state of the applied stress, from compression to neutral to tension. Fluorescence data were acquired using a Zeiss Axio Imager A2M microscope (Carl Zeiss, Inc., Jena, Germany) equipped with an AxioCAM HRm digital camera (Carl Zeiss Inc., Jena, Germany). Fluorescence excitation was provided by a 120 W Hg lamp (X-Cite 120Q, Carl Zeiss Inc., Jena, Germany). Fluorescence emission from R6G coupled to ZnO NRs was acquired using EC Epiplan-NEOFLUAR objective lens (50×, numerical aperture of 0.8) with a 2 s exposure through a spectroscopic filter cube (excitation 510–540 nm, emission 575–640 nm). For morphological characterization of the ZnO NRs, the samples were imaged using a FEI/Philips XL 20 scanning electron microscope (SEM) operating at 20 kV. Raman scattering measurements were performed on pristine ZnO NRs embedded in a PDMS matrix using a LabRAM HR Evolution Raman confocal microscope (Horiba Instruments Inc., Sunnyvale, CA, USA) with a 100× objective lens of a numerical aperture value of 0.9 (Olympus Corp., Waltham, MA, USA). A linearly polarized 532 nm laser with a power setting of 25 mW was used as an excitation source. Subsequently, Raman spectra were collected in the scan range of 50–500 cm^−1^ using an 1800 lines/mm grating and a charge-coupled-device (CCD) detector. The fluorescence and Raman data were analyzed using a suite of software such as Zeiss AxioVision, Horiba LabSpec 6, ImageJ, and OriginLab 9. 

## 3. Results and Discussion

### 3.1. Fabricating R6G-Coupled ZnO NRs Embedded in PDMS

**Figure 1:**
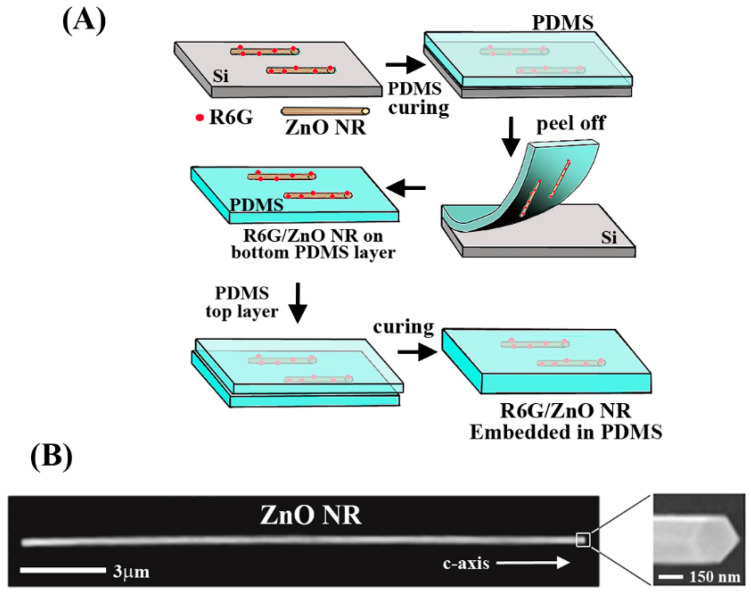
(**A**) The scheme illustrates the overall sample fabrication process for constructing R6G-coupled ZnO NRs embedded in a PDMS elastomer piece. (**B**) The SEM image displays representative ZnO NRs used in our experiments. The high shape anisotropy and crystalline features of as-grown ZnO NRs are apparent along the c-axis, as shown in the SEM image. The zoomed-in SEM panel displays the hexagonal basal facets of the NR ends and its rectangular prismic facets on its main body.

The multi-step fabrication process of R6G-coupled ZnO NRs embedded in a polydimethylsiloxane (PDMS) matrix is schematically shown in Figure 1A. By utilizing the sandwich fabrication processes detailed in the experimental section, the R6G-coupled ZnO NRs are completely encased with PDMS. This embedment process of R6G/ZnO NRs inside a PDMS matrix ensured that the NRs can be compressed and stretched using a microvice without slipping on the matrix. In order to investigate the effect of strain on the light signals derived from an extrinsic source rather than the optical signals from ZnO NR itself, R6G molecules located on the surface of the NR were excited to emit fluorescence. The emitted light signals from the dyes were then coupled into the NR optical cavity and guided along the NR. Our ZnO NRs synthesized via chemical vapor deposition do not show any emission in the visible wavelength range, providing no spectral interference with the spectroscopic profile of R6G. We note that the employment of optical quality ZnO NRs is crucial to accurately measuring the change in the NR’s fluorescence waveguiding capabilities as a function of mechanical strain applied on the NR. Representative SEM images of pristine ZnO NRs are displayed in Figure 1B. As-grown ZnO NRs exhibit a wurtzite crystal structure with six prismic facets along the NR main body and two basal facets at each of the NR ends. The zoomed-in SEM image in Figure 1B clearly shows the different crystalline facets of such wurtzite ZnO NRs. The highly anisotropic ZnO NRs studied in our experiments ranged from 5–15 µm in length by 150–300 nm in diameter.

### 3.2. NR-Guided R6G Fluorescence Intensity under Compression/Tension

**Figure 2:**
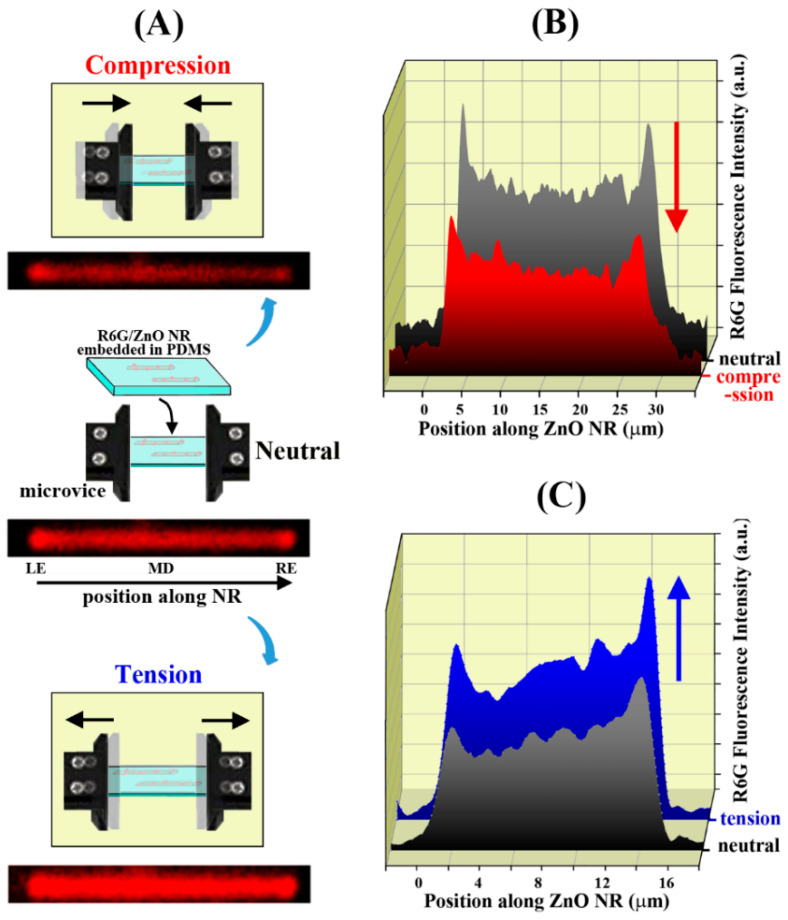
(**A**) The schematic illustrates the experimental set-up used to apply uniaxial compressive (above) or tensile (below) strain onto R6G/ZnO NRs embedded in a PDMS matrix. The red-colored fluorescence panel under each microvice setting qualitatively presents the waveguided emission profile of a R6G-coupled ZnO NR when the NR was subject to a compressive (above), neutral (center), and tensile (below) condition. (**B**) The plot quantitatively shows the change in the waveguided intensity values of R6G fluorescence measured along the NR long axis when the same ZnO NR was under a neutral versus compressive state. The red graph in the forefront is the fluorescence intensity under compression and the grey graph behind shows the fluorescence intensity without strain. The red arrow highlights the decreasing trend of the waveguided fluorescence intensity under compression. (**C**) A similar plot of R6G fluorescence intensity versus the position along the ZnO NR is displayed for tension. The blue graph in the back is for the fluorescence intensity when the NR underwent tensile strain and the grey graph in the forefront is from the neutral state. Unlike the compressive case in (**B**), an increase in the waveguided fluorescence intensity was detected when the NR was under tension, and this is highlighted by the blue arrow.

Upon applying uniaxial strain to a R6G/ZnO NRs/PDMS construct, changes in R6G fluorescence intensities along the various positions of the same NRs were monitored and compared to those of an unstrained case. Figure 2A presents such changes in R6G fluorescence intensities measured from a ZnO NR embedded in an elastomer after undergoing uniaxial compression (top), neutral (middle), and tensile (bottom) strain. Fluorescence images of the same ZnO NR are provided under the schematics showing our experimental setup for the different strain cases. Although qualitative, the fluorescence panels of the NR show an increasing trend in R6G intensity as the NR state changes from compression, to neutral, or to tension. LE, MD, and RE in Figure 2A denotes the left-end, middle, and right-end positions along the NR’s c-axis, respectively. The fluorescence intensity values of the NR were also quantitively evaluated by plotting the emission intensity versus the position along the NR. The results are displayed in Figure 2B,C for a compression and tension case, respectively. When the ZnO NR underwent compressive strain (red), the coupled fluorescence emission along the NR decreased compared to the neutral case (grey), as seen in Figure 2B. It is also clear from the graphs in Figure 2B that the application of compression causes a decrease in waveguided fluorescence intensity along all positions on the NR. In contrast, the overall intensity of the fluorescence signal increased when the same R6G/ZnO NR was under tensile strain (blue) compared to its neutral state (grey), as presented in Figure 2C. These fluorescence intensity trends are representative of a total of 115 ZnO NRs from 10 different elastomer constructs we examined.

### 3.3. Varying Levels of Uniaxial Compression on NR-Guided R6G Fluorescence Intensity

**Figure 3:**
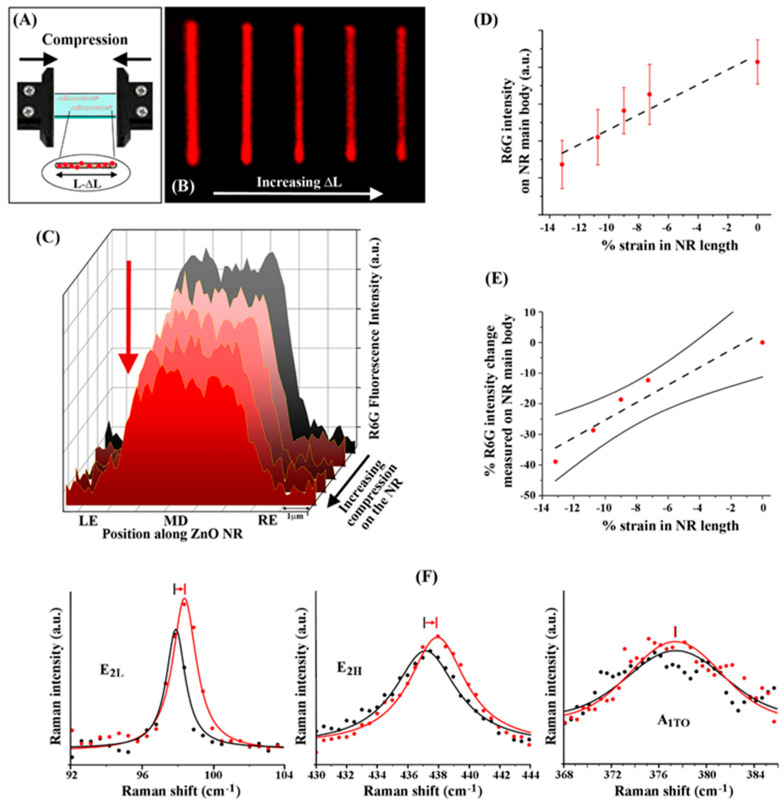
Representative R6G fluorescence intensity along individual ZnO NRs linearly decreasing with the addition of incremental compressive strain. (**A**) The schematic shows varying levels of uniaxial compressive strain being applied to R6G/ZnO NRs embedded in a PDMS matrix. The resulting NR length is depicted as L − ΔL, where L is the length of the ZnO NR at rest and ΔL is the change in length due to the compressive strain. (**B**) The compiled fluorescence panel qualitatively displays the decreasing intensity from the R6G emission on the ZnO NR with increasing ΔL, correlating to a decreasing overall NR length. (**C**) The cascading plot shows the R6G fluorescence intensity along the different position on the ZnO NR versus the levels of applied compression. The left-end, middle, and right-end positions of the ZnO NR are denoted as LE, MD, and RE, respectively. The grey graph is the farthest back of the plot shows the R6G fluorescence intensity under the neutral state of the ZnO NR. The graphs cascading towards the forefront correspond to increasing compression on the NR. The decrease in fluorescence intensity with increasing compressive strain is marked with a red arrow. (**D**) The plot displays average R6G intensity on the NR against the % strain in its length under compressive strain. The more negative values in the x-axis denote higher compression states compared to the rest state at 0% strain. The error bars correspond to the standard deviation of the 115 ZnO NRs studied under compression. (**E**) The plot shows the % R6G intensity change measured on the NR versus the % strain in NR length under compressive strain. The solid black lines above and below the data points display the 95% confidence interval. As a visual guide, the dashed lines in (**D**,**E**) are linear fits through the data points. (**F**) Raman spectra were collected of a pristine ZnO NR embedded in PDMS under no stress (black) and subsequently under compression (red) for the characteristic phonon modes of E_2H_, E_2L_, and A_1TO_ in wurtzite ZnO NRs. The observed shifts in E_2H_ and E_2L_ peaks to a higher energy along with the unchanged A_1TO_ peak confirms that the NRs in this case are under uniaxial compressive strain.

We further investigated the change in R6G fluorescence signals by systematically varying the levels of compressive strain that was uniaxially applied along the c-axis of ZnO NRs. This process is schematically depicted in Figure 3A with the associated change in NR length marked as L − ΔL. A series of fluorescence images was taken from ZnO NRs coupled with R6G whose constructs were under incremental compressive strain. Figure 3B displays a representative series of such emission panels measured from the same NRs. The laterally stacked fluorescence panels of the same NR shown in Figure 3B correspond to, from left to right, increasing compression on the NR. The images qualitatively display the trend for decreasing overall length of the NR and diminishing R6G fluorescence intensity under increasing compression. The plot of R6G fluorescence intensity versus the position on the NR in Figure 3C quantitatively shows the relationship between the waveguided R6G fluorescence intensity and the compressive strain on the NR. The cascading graphs in the plot assemble the neutral, strain-free state (grey) and four states of increasing compressive strain on the same R6G-coupled ZnO NR (pink to red). The neutral state of the ZnO NR showed the highest overall R6G fluorescence intensity, which gradually lessened with added compressive strain.

The plot in Figure 3D shows R6G intensity on the NR against the % strain in its length under compressive strain. The intensity data plotted in the graph correspond to the average intensity values of the R6G emission along the main body of the ZnO NR. It is clear from the plot that the R6G signals show a linearly decreasing relationship with higher % compressive strain. When the NR was in the neutral state at 0% strain, it showed the highest R6G fluorescence emission along the ZnO NR. With the addition of more compressive strain, i.e., higher negative values in % strain, the R6G fluorescence intensity decreased. The graph in Figure 3E displays the influence of the % strain on the R6G intensity in terms of the % change in the measured fluorescence emission. The plot further corroborates the linear behavior between the % strain and the % change in the coupled R6G emission intensity. The ZnO NR under the compressive strain of −14% resulted in approximately 35% decrease in R6G fluorescence intensity compared to its unstrained state. The behavior of decreasing fluorescence intensity due to higher % compressive strain was seen in all 115 ZnO NRs coupled with R6G analyzed in our study. 

We have further employed Raman scattering to validate that ZnO NRs indeed experience uniaxial compressive strain under the measurement setup used in our experiments. The phonon scattering peaks of E_2H_, E_2L_, and A_1TO_ of the wurtzite ZnO NR were specifically acquired, and the results are presented in Figure 3E. The two E_2_ phonon modes are sensitive to uniaxial strain along the long axis of the ZnO NR while A_1TO_ phonon mode is not affected by strain in this specific axis. In Figure 3E, the black traces in the Raman scattering data are those collected from a pristine ZnO NR embedded in PDMS whereas the red traces are the ones taken when compressive strain was subsequently applied on the NR. The A_1TO_ peak at 378 cm^−1^, as expected, did not shift. The E_2L_ and E_2H_ phonon peaks located at 97.8 cm^−1^ and 437.5 cm^−1^, respectively, showed a shift of around 0.5 cm^−1^ to the right in both panels. These observations are in agreement with the results in a Raman scattering study of individual ZnO nanowires [34,36] that reports the effects of strain on bandgap deformation potential and phonon frequency shifts, and also confirms that the NRs examined in this portion of our experiments underwent uniaxially applied compressive strain as intended.

### 3.4. Varying Levels of Uniaxial Tension on NR-Guided R6G Fluorescence Intensity

**Figure 4:**
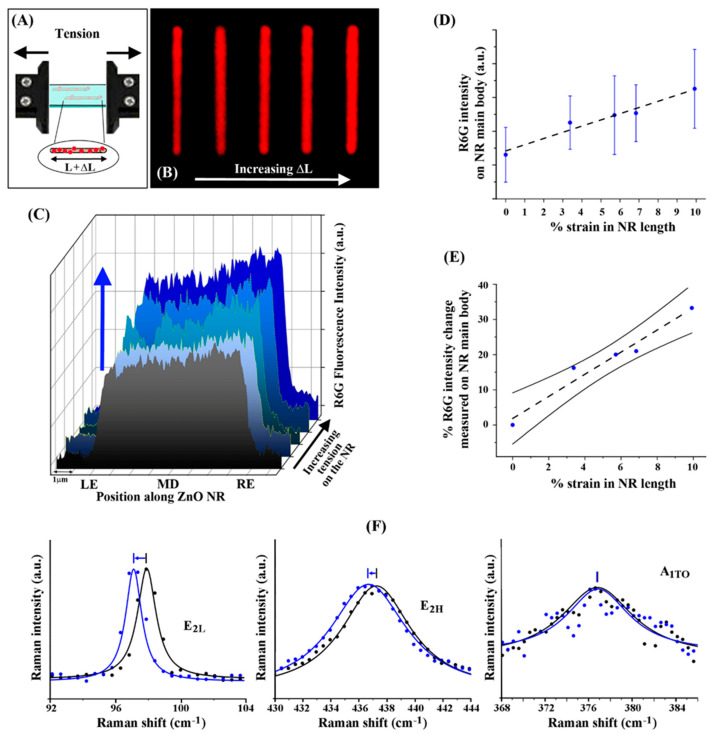
Representative R6G fluorescence intensity along individual ZnO NRs linearly increasing with the addition of incremental tensile strain. (**A**) The schematic shows varying levels of uniaxial tensile strain being applied to R6G/ZnO NRs embedded in a PDMS matrix. The resulting NR length is depicted as L + ΔL, where L is the length of the ZnO NR at rest and ΔL is the change in length due to the tensile strain. (**B**) The compiled fluorescence panel qualitatively displays increasing intensity from the R6G emission on the ZnO NR with increasing ΔL, correlating to an increasing overall NR length. (**C**) The waterfall plot shows the R6G fluorescence intensity along the ZnO NR for different levels of applied tension. The left-end, middle, and right-end positions of the ZnO NR are denoted as LE, MD, and RE, respectively. The grey graph in the front shows the R6G fluorescence intensity under the neutral state. The graphs cascading towards the back are from the cases of increasing tension on the NR. The increase in fluorescence intensity with increasing tensile strain is highlighted with the blue arrow. (**D**) The plot displays R6G intensity on the NR against the % strain in its length under tension. The R6G fluorescence intensities along the NR at rest and increasing tensile states are plotted at 0 and higher positive values of % strain. The error bars denote the standard deviation of all 115 ZnO NRs studied under tension. (**E**) The plot shows the % R6G intensity change measured on the NR versus the % strain in NR length under tensile strain. The solid black lines above and below the data points indicate the 95% confidence interval. As a visual guide, the dashed lines in (**D**,**E**) are linear fits for the data points. (**F**) Raman spectra for the phonon modes of E_2H_, E_2L_, and A_1TO_ were collected from a clean ZnO NR embedded in PDMS under no stress and subsequently under tensile strain. The strain-free and tension data are shown in black and blue, respectively. The E_2H_ and E_2L_ peak shifts to a lower energy confirms that our NRs in this portion of the study sustained uniaxial tension as intended.

With the intriguing outcomes discussed above for compression, subsequent tensile experiments on R6G/ZnO NRs were performed to provide further understanding of the relationship between the directionality in uniaxial strain and the NR-coupled R6G fluorescence intensity. The microvice illustration in Figure 4A depicts the application of tension on the R6G/ZnO NRs embedded in a PDMS matrix with the associated change in the NR length as L + ΔL. The representative series of fluorescence images in Figure 4B present an individual ZnO NR under incremental tensile strain. From left to right, the series of fluorescence panels show the increasing trend in R6G intensity with greater tension. 

The correlation between R6G fluorescence intensity and the tensile strain on the NR is shown in the waterfall plot in Figure 4C. From front to back, the ascending graphs arrange the neutral state (grey) and four states of increasing tensile strain on the same R6G-coupled ZnO NR (light blue to blue). The neutral state displayed the lowest overall fluorescence emission along the NR, whose intensity steadily rose with greater tensile strain. The plot in Figure 4D shows the average intensity values of the R6G emission along the main body of the ZnO NR as a function of % tensile strain. The application of greater tensile strain, seen with higher positive values in % strain where 0% refers to the neutral state, led to linearly increasing R6G fluorescence intensity. The graph in Figure 4E displays % R6G intensity change measured on the NR versus the % strain in NR length under tension. The result in Figure 4E further substantiates the linear trend between NR-waveguided R6G fluorescence intensity and tension. For example, comparing the two cases of 0% versus 10% strain, the added tensile stress led to a change of roughly 35% in R6G signals along the NR. This tendency of increasing fluorescence intensity associated with higher % tensile strain was seen in all 115 R6G-coupled ZnO NRs analyzed in our study. Raman spectra were also collected to validate that the ZnO NRs indeed undergoing underwent uniaxial tensile stress, and the results are presented in Figure 4F. Similar to the compression case in Figure 3F, neutral state spectra were initially procured from a clean ZnO NR embedded in PDMS and the measurements were repeated while the NR was under tension. The A_1TO_ phonon mode did not show any peak shift, whereas the E_2H_ and E_2L_ phonon peaks were shifted to the left by approximately 0.7 cm^−1^, confirming that our NRs in this portion of the study sustained uniaxial tensile stress as intended.

### 3.5. Effect of Uniaxial Strain on FINE and DoF

**Figure 5:**
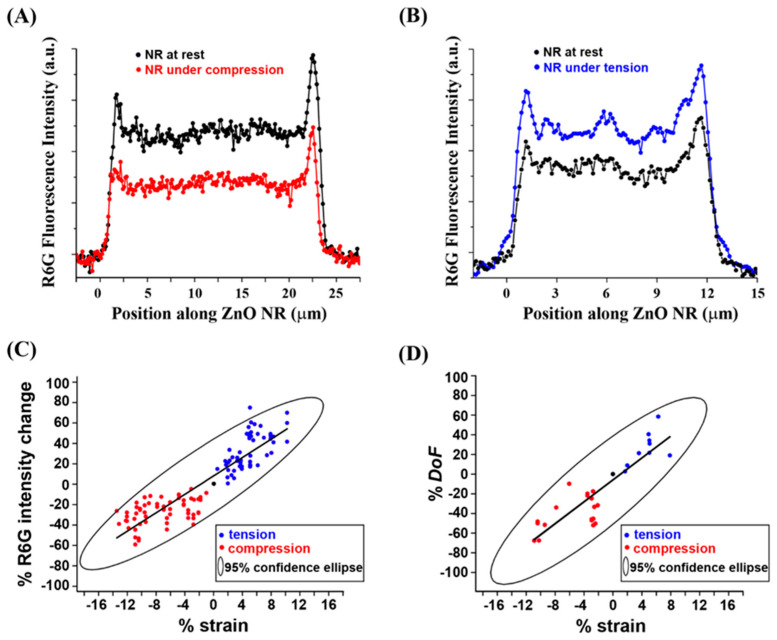
The change in DoF in % R6G fluorescence intensity was correlated to that of % strain by performing systematic measurements under a series of compression/tension and obtaining data from the end and middle positions on individual ZnO NRs. (**A**,**B**) The plots display R6G fluorescence intensity as a function of position along the same ZnO NR when the NR was under compression (**A**) and tension (**B**). Black, red, and blue data points correspond to the NR cases of neutral, compression, and tension, respectively. (**C**) The plot shows the relationship between the % strain compiled from compression to tension and the % R6G intensity values measured on both the end and middle positions of ZnO NRs. (**D**) The plot presents the compiled outcomes of the R6G fluorescence intensity measured in terms of % DoF with respect to the amounts of % compressive/tensile strain applied to individual ZnO NRs. The solid black line and circle in (**C**,**D**) are a linear fit through the data and the 95% confidence ellipse, respectively. The negative signs indicated on the tick labels of the graphs in (**C**,**D**) simply refer to the measurement case of compression.

As discussed earlier, our previous studies have shown that individual ZnO NRs efficiently waveguide coupled light through the NR main body to the two ends of the NR which, in turn, leads to highly localized and intensified signals at the NR ends. This effect known as FINE can be evidenced by the highly intensified R6G fluorescence signals at the two NR end positions in the plots in Figure 5A,B. In order to understand the influence of compressive and tensile strain and their effect on the waveguiding properties of ZnO NRs, a ratio between the average fluorescence intensity measured on the NR main body relative to that measured on the NR ends was compared. The mechanical strain-dependent results in the degree of fluorescence intensification on the NR ends can provide a quantitative means for ascertaining the extent of the effect of mechanical stress on the NR has on its waveguiding ability of the R6G fluorescence emission through the NR cavity. The R6G fluorescence profiles along single ZnO NRs shown in Figure 5A,B are representative of the position-dependent emission intensities used to calculate the degree of FINE (DoF) values, i.e., DoF = (I_avg,end_ − I_avg,mid_)/I_avg,mid_. I_avg_ is the average fluorescence intensity and the subscript of end/mid refers to the end/middle positions of the NR. Regardless of the NR position analyzed, a linear relationship was found between % strain and % R6G fluorescence intensity along the ZnO NR, as seen in Figure 5C. Relative to the measured fluorescence intensity under a neutral state, compression decreased the coupled fluorescence intensity on the NR, with higher compressive strain leading to reduced fluorescence intensity. In contrast, larger % tensile strain yielded greater % R6G intensity. Similarly, a linear relationship was revealed in Figure 5D between the % strain and % R6G intensity in % DoF. Uniaxially applied tensile stress on the NR leads to a greater DoF, i.e., more efficient guiding of the R6G fluorescence signals to the NR ends via the NR optical cavity, than a compressive stress case. Higher magnitudes of tensile (compressive) stress on the NR yields a larger (smaller) DoF value.

It is known that the refractive index of the waveguide can influence the amount of light that couples into the optical cavity, where an increase in the refractive index provides more efficient light coupling into the guiding medium and further waveguiding of the light through the cavity via total internal reflection. The observed trend of strain versus fluorescence intensity in our experiments may be caused by the fact that the application of compressive (tensile) stress on the ZnO NRs led to a decrease (increase) in the NR’s refractive index which, in turn, resulted in a lower (higher) R6G fluorescence signal. This change in refractive index will also influence DoF, as a strain condition yielding a higher refractive index can enable more coupling of light waveguided towards the NR ends. Insights from previous studies involving the NR’s intrinsic emission properties indicated that, under tension (compression), a smaller (larger) bandgap is measured relative to an unstrained case [30,31,32,33]. In other studies, an inverse relationship between the bandgap and refractive index of a semiconducting material has been shown [37,38]. In addition, a recent computational study on planar waveguides have correlated the relationship of compressive (tensile) strain to a decrease (increase) in refractive index [39]. Other studies based on finite element modeling (FEM) and 3D finite-difference time-domain (FDTD) have also shown the effects of bending strain to the electric and magnetic field intensities on ZnO nanowires [40,41]. These studies concluded that mechanical strain experienced by the nanowires and subsequent changes in refractive index of the nanomaterials can lead to a phase shift of transmitted light, optical path of light, and fraction of incident light [40,41]. These works seem to indirectly suggest that the trend observed in our experiments may be attributed to the changes in the NR refractive index under the different conditions of strain exerted on the NRs. Moreover, valuable insights from existing modeling and computation studies on gold nanospheres and nanorods [42] point to an exciting opportunity that strain-induced changes in local electromagnetic fields and ensuing enhancement/suppression of ZnO nanomaterials’ optical responses may be controlled by factors such as the length and radius of ZnO NRs. Further investigations are on-going in order to systematically determine the exact origin of the observed trend of strain versus fluorescence intensity in our fluorophore-coupled NR systems.

## 4. Conclusions

In summary, we systematically investigated the effect of uniaxial compressive and tensile strain on the fluorescence intensity emitted by R6G, whose light was subsequently coupled to and guided by individual ZnO NRs. We ascertained that compression (tension) leads to a decrease (increase) in R6G fluorescence signal on ZnO NRs. Compared to an unstrained state, approximately 35% decrease (increase) in R6G fluorescence intensity was observed from ZnO NRs when they were under compressive strain of −14% (tensile strain of +10%). By examining NRs undergoing incremental levels of compressive (tensile) strain, we elucidated a linear relationship between compression (tension) and decreased (increased) fluorescence intensity. Additionally, through the position-dependent analysis on single NRs, we revealed the effect of strain on DoF in coupled R6G fluorescence intensity, which also corresponded to a linear relationship. We successfully demonstrated that different types and levels of strain can be effectively used to modulate the waveguiding characteristics of single ZnO NRs in terms of guiding optical signals from an extrinsic source nearby. Hence, our efforts may be valuable for implementing strain as an effective tool to control and expand the applications of single ZnO NRs such as those for nanoprobes enabling localized light delivery with nanoscale precision and for fluorescence-based detection platforms for chemical and biological analytes.
